# Modulation of pain perceptions following treadmill running with different intensities in females

**DOI:** 10.14814/phy2.15831

**Published:** 2023-09-25

**Authors:** Xu Zi‐Han, An Nan, Chang Jeremy Rui, Yang Yong‐Long

**Affiliations:** ^1^ School of Sport Medicine and Rehabilitation Beijing Sport University Beijing China; ^2^ Department of Rehabilitation Sciences The Hong Kong Polytechnic University Hong Kong China

**Keywords:** analgesic effects, endogenous pain modulation, exercise‐induced hypoalgesia, pain thresholds, pain tolerance thresholds

## Abstract

We aimed to compare the effects of three intensities of treadmill running on exercise‐induced hypoalgesia (EIH) in healthy individuals. We anticipated that the primary and secondary changes in pain perception and modulation may differ between running intensities. Sixty‐six women were randomly assigned to one of three treadmill running intensities for 35 min: 40% reserved heart rate (HRR), 55% HRR, or 70% HRR. The effects of EIH were assessed using pressure pain thresholds (PPT) and tolerance thresholds (PPTol). We measured conditional pain modulation (CPM). Compared with baseline, PPT and PPTol significantly increased in all groups during running and at the 5–10‐min follow‐up. The PPT and PPTol changes in the moderate‐ and low‐intensity groups were significantly higher than those in the high‐intensity group during running and 24 h after running, while the CPM responses of the high‐intensity group were significantly reduced at the 24‐h follow‐up. Moderate‐ and low‐intensity running may elicit significant primary and secondary (persisting over 24 h) EIH effects and increase CPM responses in females. However, high‐intensity running induced only limited analgesic effects and reduced CPM responses, which may be attributed to the activation of endogenous pain modulation.

## 1 INTRODUCTION

Exercise has been identified as an effective intervention for managing patients with pain syndromes. Previous studies have demonstrated that global aerobic exercises (Vaegter et al., 2018) or local resistance exercises (Harris et al., 2018) can effectively alleviate pain perception (Rice et al., 2019) and enhance emotional well‐being, commonly called exercise‐induced hypoalgesia (EIH; Vaegter & Jones, 2020). Endogenous pain modulation (Bobinski et al., 2015) and the cortical cognitive process of pain (Holmes et al., 2021) are associated with EIH. Exercise can trigger endogenous descending inhibition of nociceptive signals from the dorsal horn of the spinal cord (Tong et al., 2021) and activate the reward circuits of the corticolimbic systems (Kami et al., 2020).

The analgesic effect following exercise in asymptomatic individuals tends to be correlated with exercise intensities (Baiamonte et al., 2017; Naugle, Naugle, Fillingim, Samuels, & Riley 3rd., 2014), while pain tolerance thresholds can also significantly improve during various physical activities (de Zoete et al., 2020). However, an individual's EIH can be affected by endogenous pain modulation (Fingleton et al., 2017; Naugle & Riley 3rd., 2014) or psychological factors (Naugle, Naugle, Fillingim, & Riley 3rd., 2014) such as pain catastrophizing, fear, anxiety, or depression. Notably, high‐intensity exercises (>6 metabolic equivalents, METs) often fail to restore pain detection thresholds in individuals with pain conditions (Van Oosterwijck et al., 2010), while low‐ or moderate‐intensity activities (3–6 METs) can increase pain tolerance thresholds (Newcomb et al., 2011).

The EIH effects following exercises of different intensities might be attributed to the mechanisms of endogenous pain modulation (Koltyn et al., 2014), which have different activation thresholds for the perception stimulus (You et al., 2022). The ventromedial nucleus of the thalamus can be activated by inputs from both noxious and non‐noxious C fibers, which can be triggered via muscle contraction (Adreani et al., 1997) and can induce descending inhibition. The thalamic mediodorsal nucleus can trigger descending facilitation via input from noxious C fibers. The periaqueductal gray (PAG; Lei et al., 2014) and rostral ventromedial medulla (RVM; Fields et al., 1995) can modulate nociception signals by projecting to the dorsal horn of the spinal cord and changing the pressure pain detection (PPT) and tolerance threshold (PPTol).

Given that the threshold of descending facilitation is lower than that of inhibition (You et al., 2013), the activation of pain inhibition usually requires a high‐intensity painful stimulus (conditioned stimulus) called conditioned pain modulation (CPM). Thus, high‐intensity exercise may activate the opioid (Kim et al., 2015) systems in the PAG and RVM (Brito et al., 2017), inducing pain inhibition, but not in individuals with impaired CPM function (Vaegter et al., 2021). High‐intensity exercise may also trigger descending facilitation (Alsouhibani et al., 2019), which may decrease EIH and CPM responses, leading to mechanical allodynia (Sluka et al., 2012), producing delayed onset muscle soreness (DOMS; Dannecker et al., 2002) in asymptomatic individuals (Kruger et al., 2016).

However, moderate‐ or low‐intensity stimuli may activate the cannabinoid (Crombie et al., 2018) and 5‐hydroxytryptamine (5‐HT; Bobinski et al., 2015) systems, inducing descending pain inhibition via the temporal summation (You et al., 2014) of non‐noxious C‐fiber inputs without triggering nociceptors and descending facilitation. The inhibition‐only EIH effects following moderate‐ or low‐intensity exercise may not induce secondary mechanical allodynia or DOMS, which has not yet been verified in human studies.

Considering the potential modulation of EIH effects by exercises of different intensities, we aimed to compare the primary and secondary changes in PPT and PPTol in asymptomatic individuals following running exercises of various intensities. We also measured changes in CPM responses before and 24 h after the running sessions to preliminarily reveal the role of endogenous pain modulation in EIH effects.

We hypothesized that (1) low‐, moderate‐, and high‐intensity running exercise might elicit EIH responses and increase the PPT and PPTol, (2) the analgesic effects of moderate‐ or low‐intensity exercise may persist at the 24‐h follow‐up, while high‐intensity exercise may decrease the pain threshold the next day, and (3) the CPM responses at 24 h following high‐intensity running might be attenuated compared with those following low‐ and moderate‐intensity running.

## 2 METHODS

This study was approved (2023023H) by the Sports Science Experimental Ethics Committee of Beijing Sport University.

### 2.1 Study design

Sixty‐nine healthy participants were included in this study and invited to perform exercise interventions of different intensities. Informed consent forms were provided and signed by all participants before participating in this study. Demographic data and baseline measurements (such as resting heart rate [HRrest], PPT, PPTol, and CPM responses) were collected. The maximum heart rate (HRmax) was estimated using the formula (Lach et al., 2021): HRmax = 202.5–0.53 × age, and the reserved heart rate (HRR) was calculated as HRR = HRmax−HRrest. Real‐time HR was collected and recorded via the HR belt worn by the participants during running. To avoid the potential long‐lasting analgesic effects of the CPM test, all exercise interventions were performed 1 week after the baseline measurements.

All participants conformed to the study and were randomly assigned to three experimental groups (A, B, and C) with different exercise intensities in a 1:1:1 ratio. Randomized sequences were generated using Excel software (Microsoft). All participants were labeled from 01 to 66 and allocated according to the A–B–C circulation order. AN and XZH screened the participants.

**FIGURE 1:**
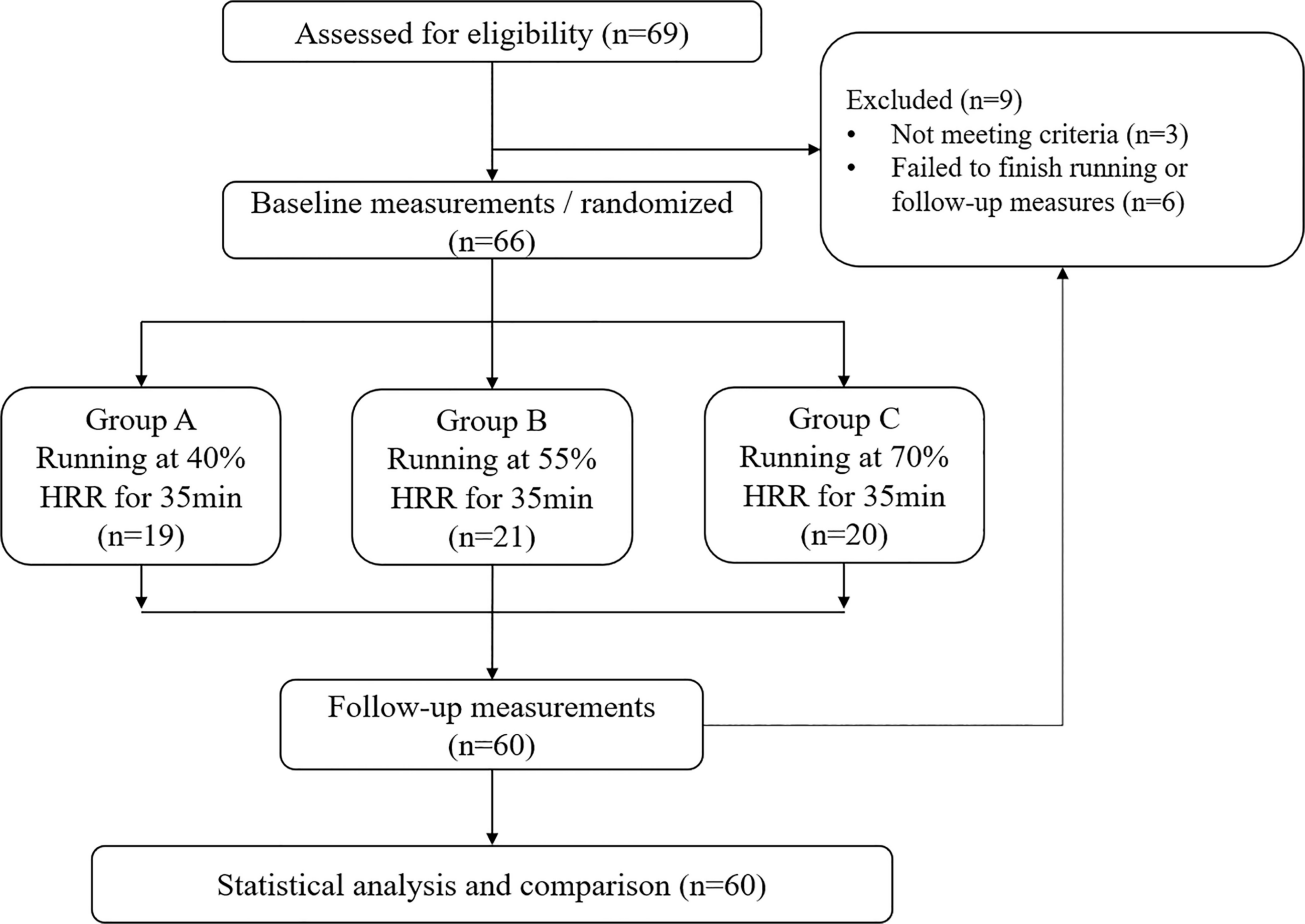
Flowchart of the experiment.

The participants performed low‐intensity treadmill running with 40% HRR in group A, moderate‐intensity (55% HRR) in group B, or high‐intensity (70% HRR) in group C. The running speed was determined in coherence with the target heart rate (THR) during the baseline measurements. All the participants performed a single exercise session at a predetermined intensity for 35 min (Figure 1).

### 2.2 Participants

Based on previous studies (Hviid et al., 2019; Pacheco‐Barrios et al., 2020), aerobic exercise‐induced effect size on PPT changes ranged from 0.20 to 0.38, and PPTol was 0.20. Our study utilized G*Power software with an effect size of 0.38, an alpha level of 0.05, and a power of 0.80. Thus, a minimum sample size of 66 participants across the three groups was determined.

Sixty‐nine healthy female students (aged 18–30 years) from Beijing Sport University were included in this study, 66 of whom were enrolled. The exclusion criteria were: (1) pain‐related pathological or psychological syndrome within 3 months; (2) injury history of lower extremities within 1 year; (3) potential or confirmed heart disease or recovery from a heart disease less than 1 year; (4) failure to maintain or tolerate the exercise intensity during the treadmill running; (5) serious exertion or fatigue 24 h after exercise sessions; (6) intolerable pain during the pain perception test or the CPM test; (7) current menstruating; and (8) regular exercise or previous training experiences.

### 2.3 Procedures

**FIGURE 2:**
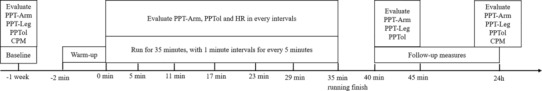
Flowchart of the procedures.

All participants performed a single treadmill running session at different intensities based on their THR. The THR was 40% HRR in group A, 55% in group B, and 70% in group C. The participants wore an HR belt to monitor and record the real‐time HR during the test and running sessions. A running assessment was administered to every participant 1 week before implementing the exercise intervention. This assessment involved a progressive increase in speed until the THR was reached. Subsequently, the predetermined speed for each individual was established at the commencement of running. During the running session, the running speed was adjusted at any time based on the participant's HR changes (Figure 2).

Baseline PPT and PPTol were measured 10 min before the exercise session, and CPM responses were tested to mitigate the possibility of long‐lasting analgesic effects. The exercise session consisted of 35 min of running with five interval periods (at a speed of 4 km/h for 1 min) every 5 min. Throughout each interval, HR, PPT‐arm, and PPTol were measured. Post‐exercise PPT and PPTol were conducted 5 and 10 min after exercise. Additionally, PPT, PPTol, and CPM were measured 24 h after the exercise session.

### 2.4 Outcome measures

Outcome measures were assessed at multiple time points: before, during, and after the running session. The PPT‐arm and PPTol were recorded at every interval during running, and the PPT‐leg was only tested after the running session. CPM responses were evaluated using cold pressure methods at baseline and 24 h after the running session. All testing locations were marked with a sterile waterproof marker to ensure consistency in the repeated measures. The testing angle of the algometer was carefully adjusted perpendicular to the skin.

### 2.5 Pressure pain threshold

Pressure pain threshold was evaluated using a quantitative sensory testing protocol (Wytrazek et al., 2015) with a handheld pressure algometer (Baseline Dolorimeter, Fabrication Enterprises) equipped with a 1‐cm (Harris et al., 2018) metal probe. Pressure was applied at a rate of 0.5 kg/s over two locations: the extensor carpus radialis (PPT‐arm) and peroneus longus (PPT‐leg) on the right side. The participants were instructed to indicate their perceived pain intensity using the visual analog scale (VAS) ranging from 0 to 100. When participants reported a pain intensity of 30 out of 100 (Pain30) during pressure application, the pressure thresholds were recorded as PPT values.

### 2.6 Pressure pain tolerance threshold

Pressure pain tolerance threshold was assessed using a quantitative sensory testing protocol (Bellomo et al., 2020) via a handheld pressure algometer (Baseline Dolorimeter, Fabrication Enterprises) with a 1‐cm (Harris et al., 2018) metal probe. Pressure was applied at 0.5 kg/s over the extensor carpus radialis on the left side. The participants were instructed to indicate their perceived pain intensity using the VAS ranging from 0 to 100. When the participants reported a pain intensity of 70 out of 100 (Pain70) during pressure application, the pressure threshold was recorded as the PPTol value.

### 2.7 Conditional pain modulation

The CPM response was measured using a quantitative sensory testing protocol, specifically the cold pressor procedure (Coulombe‐Leveque et al., 2021). In this procedure, pressure was applied as the test stimulation, and cold water immersion served as the conditioned stimulation. Participants first received pressure stimulation at the ipsilateral extensor carpus radialis and reported the PPT as a test stimulus when the pain intensity reached Pain30. Subsequently, participants were instructed to immerse the contralateral hand into cold water at 8°C for 1 min. The PPT at Pain30 was reassessed when the participants withdrew their hands from immersion. The difference between the two PPTs was recorded as a response to the CPM.

### 2.8 Statistical analysis

The normality of all data was assessed using the Shapiro–Wilk test. Differences in baseline data (height, weight, HRrest, CPM, PPT, and PPTol) between the groups were analyzed using one‐way analysis of variance (ANOVA). To determine the differences among the three groups over time (running times and acute follow‐up times), a two‐way (running time and intensity) repeated‐measures ANOVA was applied to examine PPT and PPTol, except for the PPT‐leg at 35 min, which was tested via one‐way analysis of covariance (ANCOVA) with the baseline measurements set as covariates. For changes in PPT and PPTol 24‐h post‐running, one‐way ANCOVA was also applied. Changes in CPM responses were evaluated using a one‐way ANCOVA, while baseline measurements were set as covariates. Post hoc multiple comparisons were performed using the Bonferroni method. All statistical analyses were performed using SPSS Version 21.0, and a significance level of *p* < 0.05 was applied to all tests.

## 3 RESULTS

### 3.1 Baseline and running characteristics

Three participants were excluded from this study because shoulder pain syndrome occurred 1 month before the experiment. Of the 66 participants enrolled in this study, 19 in group A completed low‐intensity running for 30 min, 21 in group B completed moderate‐intensity running for 30 min, and 20 in group C completed high‐intensity running for 30 min. Six participants withdrew from the study because of onset of menstruation, failing to finish the running, and being lost to follow‐up. No significant differences were observed in baseline characteristics between the groups (*p* > 0.05, Table 1). Significant differences were observed in the HR for every interval between the groups during the running period (Table 1).

**TABLE 1:** Baseline and running measurement mean ± standard deviation (M ± SD)^a^.

Measurements	A (*n* = 19)	B (*n* = 21)	C (*n* = 20)	*p*
Age (years)	21.63 ± 2.01	22.52 ± 2.06	21.10 ± 2.20	0.097
Height (cm)	166.95 ± 7.31	166.48 ± 7.27	165.55 ± 4.93	0.797
Weight (kg)	59.05 ± 9.67	56.05 ± 9.23	57.55 ± 8.02	0.576
PPT‐arm (kg/cm^2^)	2.31 ± 0.16	2.33 ± 0.13	2.32 ± 0.17	0.949
PPT‐leg (kg/cm^2^)	4.40 ± 0.53	4.51 ± 0.48	4.46 ± 0.48	0.799
PPTol (kg/cm^2^)	4.57 ± 0.42	4.68 ± 0.49	4.61 ± 0.40	0.740
CPM (kg/cm^2^)	0.70 ± 0.15	0.70 ± 0.16	0.71 ± 0.17	0.998
HRrest (beats/min)	81.11 ± 0.89	82.71 ± 6.86	81.95 ± 7.79	0.814
THR (beats/min)	125.26 ± 5.32	142.03 ± 3.07	158.51 ± 2.40	<0.001
HR of 5 min (beats/min)	125.85 ± 5.75	143.95 ± 5.96	161.00 ± 4.67	<0.001
HR of 11 min (beats/min)	126.36 ± 6.01	143.38 ± 6.67	163.35 ± 3.92	<0.001
HR of 17 min (beats/min)	126.00 ± 6.45	142.62 ± 5.69	164.00 ± 4.34	<0.001
HR of 23 min (beats/min)	125.32 ± 6.80	143.95 ± 5.98	165.01 ± 4.52	<0.001
HR of 29 min (beats/min)	126.11 ± 6.43	143.71 ± 4.66	165.95 ± 3.79	<0.001
HR of 35 min (beats/min)	126.47 ± 5.86	144.14 ± 5.34	166.15 ± 4.15	<0.001

*Note*: All data were presented as mean ± standard deviation.

Abbreviations: CPM, conditional pain modulation; HR, heart rate; PPT, pressure pain threshold; PPTol, tolerance thresholds; THR, target heart rate.

^a^ One‐way ANOVA, significant difference was set by *p* ≤ 0.05.

### 3.2 Changes in pressure pain threshold of the arm following running

Two‐way repeated‐measures ANOVA revealed significant main effects (*F* = 264.74, *p* < 0.001) of running time on the PPT of the arm, which indicated that running for 30 min significantly increased the global PPT. The interaction effect (*F* = 13.55, *p* < 0.001) between the running intensity and time on the PPT of the arms was also significant. Post hoc comparisons revealed that the changes in PPT in the moderate‐intensity group were significantly higher than those in the low‐ (*p* = 0.003) and high‐intensity (*p* < 0.001) groups. Furthermore, low‐intensity running resulted in a higher PPT (*p* < 0.001) than high‐intensity running.

Two‐way repeated‐measures ANOVA revealed significant main effects (*F* = 385.83, *p* < 0.001) of running time on the PPT of the arm immediately after running. The interaction effect (*F* = 25.35, *p* < 0.001) of running intensity and time on the PPT of the arm was also significant. Post hoc comparisons revealed that the changes in PPT in the high‐intensity group were significantly lower (*p* < 0.001) than those in the other two groups. However, no significant differences were observed between the low‐ and moderate‐intensity groups (*p* = 0.09) in terms of the PPT changes.

**FIGURE 3:**
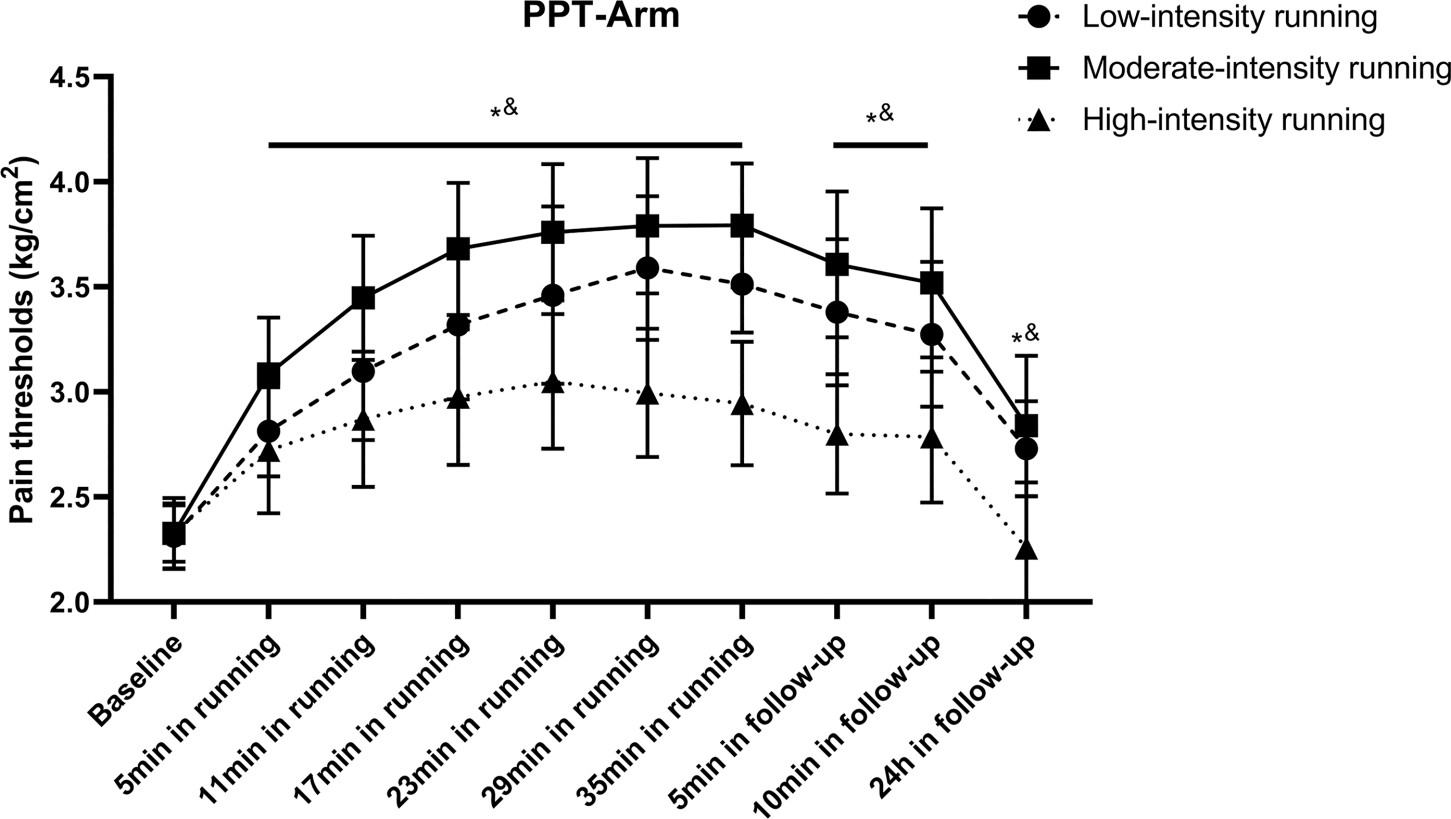
Changes in PPT of arms following running. All data were presented as mean and standard deviation; PPT, pressure pain threshold. *PPT in low‐intensity group significantly higher than high‐intensity group. &PPT in moderate‐intensity group significantly higher than high‐intensity group.

One‐way ANCOVA revealed significant between‐group differences (*F* = 16.52, *p* < 0.001) at the 24‐h follow‐up when running intensities were considered. Post hoc comparisons revealed that the changes in PPT in the high‐intensity group were significantly lower (*p* < 0.001) than those in the other two groups; however, no significant differences were observed between the low‐ and moderate‐intensity groups (*p* = 0.80; Figure 3 and Tables S1 and S2 in the supplemental files).

### 3.3 Changes in pressure pain threshold of the leg following running

Two‐way ANCOVA revealed significant between‐group differences (*F* = 19.25, *p* < 0.001) in the PPT of the legs immediately after running. Post hoc comparisons revealed that the changes in PPT in the high‐intensity group were significantly lower than those in the low‐ (*p* = 0.002) and moderate‐intensity (*p* < 0.001) groups. However, no significant differences were observed between the low‐intensity and moderate‐intensity exercise groups.

Two‐way repeated‐measures ANOVA revealed significant main effects (*F* = 181.03, *p* < 0.001) of the measurement time on the PPT of the legs 5–10 min after running. The interaction effect (*F* = 18.65, *p* < 0.001) of running intensity and time on leg PPT was also significant. Post hoc comparisons revealed that the changes in PPT in the high‐intensity group were significantly lower than those in the low‐(*p* = 0.007) and moderate‐intensity (*p* < 0.001) groups.

**FIGURE 4:**
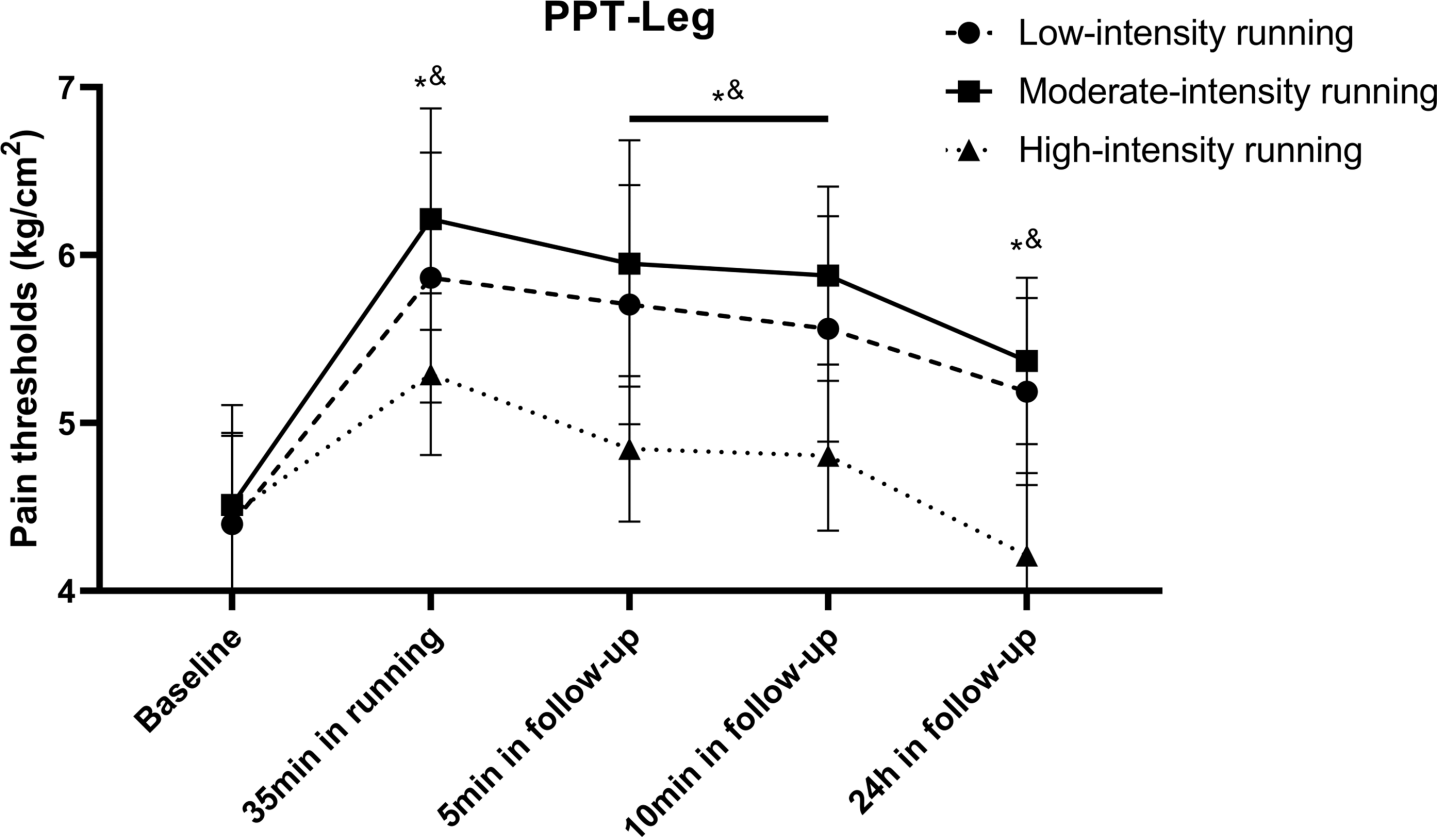
Changes of PPT in legs following running. All data were presented as mean/standard deviation; PPT, pressure pain threshold. *PPT in low‐intensity group significantly higher than high‐intensity group. ^&^PPT in moderate‐intensity group significantly higher than high‐intensity group.

One‐way ANCOVA revealed significant between‐group differences (*F* = 38.11, *p* < 0.001) at the 24‐h follow‐up when running intensities were considered. Post hoc comparisons revealed that the changes in PPT in the high‐intensity group were significantly lower (*p* < 0.001) than those in the other two groups; however, no significant differences were observed between the low‐ and moderate‐intensity groups (*p* > 0.999; Figure 4 and Tables S3 and S4 in the supplemental files).

### 3.4 Changes in pressure pain tolerance threshold following running

Two‐way repeated‐measures ANOVA revealed significant main effects (*F* = 122.93, *p* < 0.001) of running time on PPTol, which indicated that running for 30 min significantly increased PPTol. The interaction effect (*F* = 3.90, *p* < 0.001) between running intensity and running time on PPTol was also significant. Post hoc comparisons revealed that the changes in PPTol in the moderate‐intensity exercise group were significantly higher (*p* = 0.008) than those in the high‐intensity exercise group. However, no significant differences were observed between the low‐ and high‐intensity exercise groups (*p* = 0.129) or between the low‐ and moderate‐intensity exercise groups (*p* = 0.256).

Two‐way repeated‐measures ANOVA revealed significant main effects (*F* = 150.96, *p* < 0.001) for the measurement time on PPTol 5–10 min after running. The interaction effect (*F* = 4.18, *p* = 0.02) between running intensity and running time on PPTol was also significant. Post hoc comparisons revealed that the changes in PPTol in the moderate‐intensity exercise group were significantly higher (*p* = 0.006) than those in the high‐intensity exercise group. However, no significant differences were observed between the low‐ and high‐intensity exercise groups (*p* = 0.140) or between the low‐ and moderate‐intensity exercise groups (*p* = 0.199).

**FIGURE 5:**
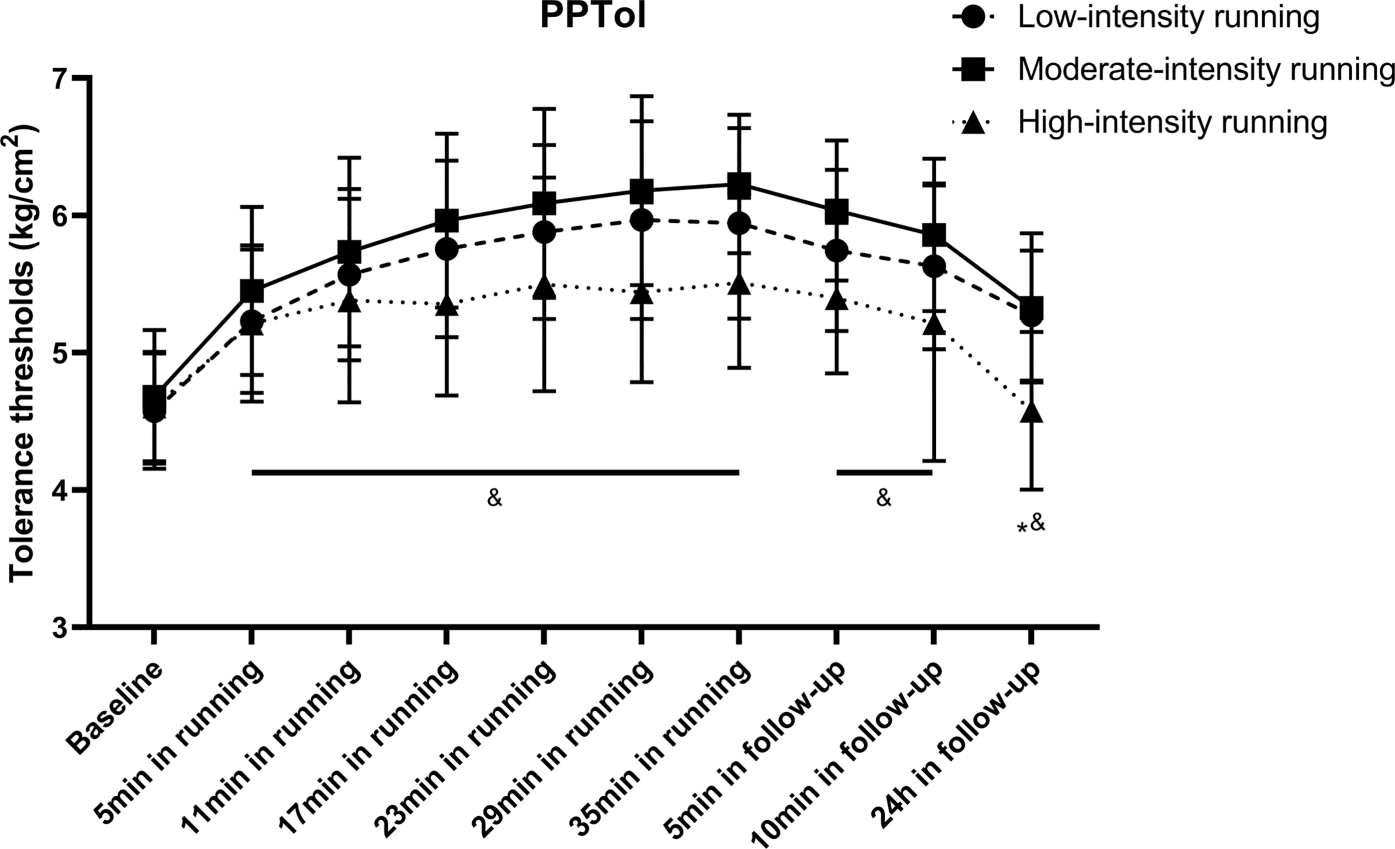
Changes in PPTol following running. All data were presented as mean/standard deviation; PPTol, pressure pain tolerance threshold. *PPTol in low‐intensity group significantly higher than high‐intensity group. ^&^PPTol in moderate‐intensity group significantly higher than high‐intensity group.

One‐way ANCOVA revealed significant between‐group differences (*F* = 13.58, *p* < 0.001) at the 24‐h follow‐up when running intensities were considered. Post hoc comparisons revealed that the changes in PPTol in the high‐intensity group were significantly lower (*p* < 0.001) than those in the other two groups; however, no significant differences were observed between the low‐ and moderate‐intensity groups (*p* > 0.999; Figure 5 and Tables S5 and S6 in the supplemental files).

### 3.5 Changes in conditional pain modulation following running

**FIGURE 6:**
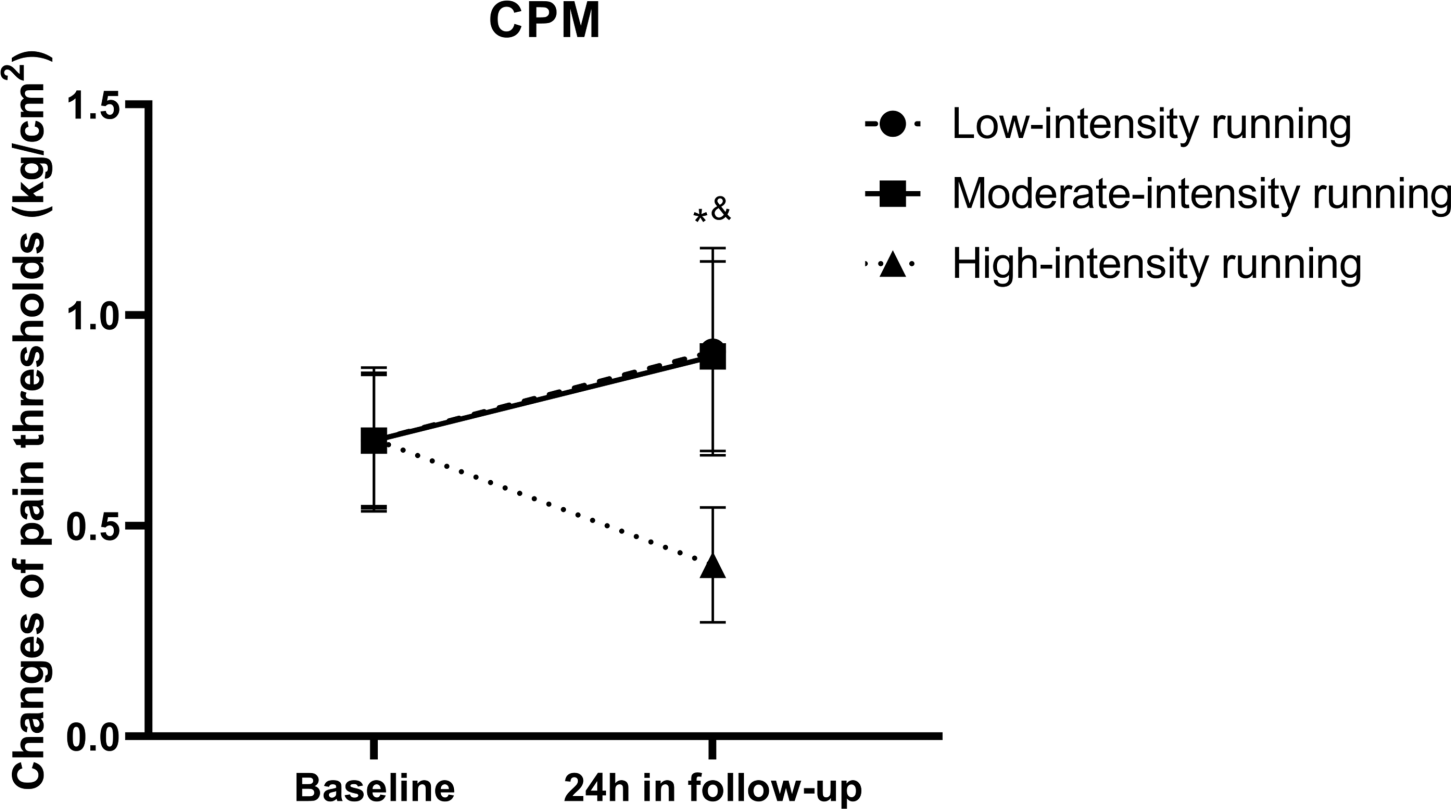
Changes in CPM following running. All data were presented as mean/standard deviation; CPM, conditioned pain modulation. *CPM in low‐intensity group significantly higher than high‐intensity group. ^&^CPM in moderate‐intensity group significantly higher than high‐intensity group.

One‐way ANCOVA revealed significant between‐group differences (*F* = 27.17, *p* < 0.001) at the 24‐h follow‐up when running intensities were considered. Post hoc comparisons revealed that the changes in CPM in the high‐intensity group were significantly lower (*p* < 0.001) than those in the other two groups; however, no significant differences were observed between the low‐ and moderate‐intensity groups (*p* > 0.999; Figure 6 and Tables S7 and S8 in the supplemental files).

## 4 DISCUSSION

We investigated the changes in pain perception following running exercises in healthy individuals. Our results revealed that the changes in PPT and PPTol increased with running time. The PPT and CPM responses to moderate‐ and low‐intensity running were significantly higher than those to high‐intensity exercise during the running sessions and follow‐ups. Furthermore, improvements in PPTol after moderate‐intensity running were significantly greater than those after high‐intensity running. These results indicate that the modulation of the effects of EIH may involve distinct central mechanisms influenced by the context of exercise.

Previous studies have examined similar changes in PPT of exercised and non‐exercised limbs following exercise with different intensities. For instance, Hoffman et al. (2004) compared PPT changes during running and discovered that running with 75% maximal oxygen consumption (VO_2_max) elicited greater EIH effects than running with 50% VO_2_max in both the hands and legs. The global analgesic effect of moderate‐intensity aerobic exercise has also been investigated in previous studies (Ellingson et al., 2014; Malfliet et al., 2018), which reported the attenuation of heat pain perception following 10 min of cycling. However, Kruger et al. (2016) observed that maximal endurance cycling induces global mechanical allodynia in the chest and head. These results indicate that the effect of EIH may not increase with exercise intensity. However, most of these studies only evaluated acute EIH effects and did not investigate secondary changes 24 h following running, which were insufficient to reveal the role of endogenous pain modulation in EIH effects. Additionally, the activation of central endogenous pain modulation may induce global effects in hypoalgesia (Gomolka et al., 2019) or hyperalgesia (Staud et al., 2005), which may explain the similar changes in PPT between the arm and leg following treadmill running.

**FIGURE 7:**
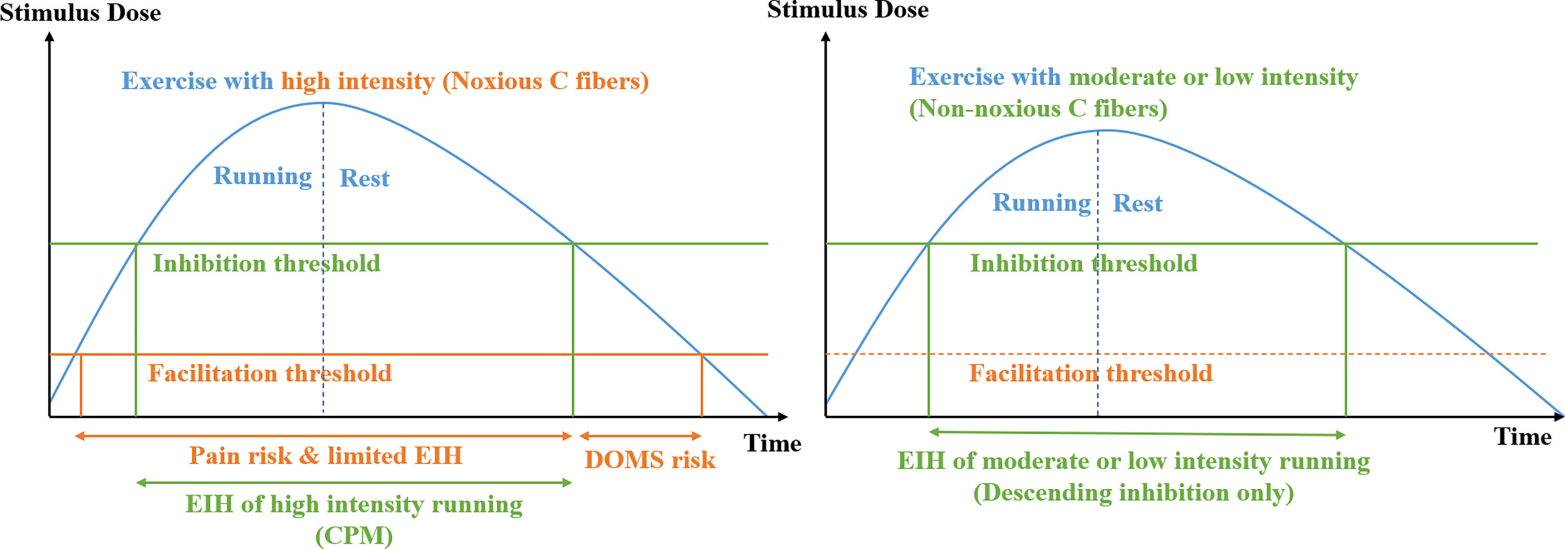
Potential mechanisms of EIH induced via running with different intensities. CPM, conditioned pain modulation; DOMS, delayed onset muscle soreness; EIH, exercise‐induced hypoalgesia.

The effects of EIH following exercises of various intensities may be introduced by the interaction between muscle contraction and C‐fiber inputs during treadmill running. Muscle contraction can activate C fibers during exercise (Adreani et al., 1997). Thus, high‐intensity exercises may trigger descending inhibition and upregulate opioids (Mazzardo‐Martins et al., 2010; Saanijoki et al., 2018) in the PAG. However, it can also activate noxious C fibers, potentially inducing descending facilitation and decreasing the EIH and CPM responses. Moderate‐ and low‐intensity exercises with sufficient duration may stimulate non‐noxious C fibers, triggering the activation of cannabinoid (Hughes & Patterson, 2020) and 5‐HT (Bobinski et al., 2015; Tour et al., 2017) receptors in the PAG and RVM. This activation can enhance CPM responses and analgesic effects (Figure 7).

The role of CPM responses on the effects has also been investigated by Lemley et al. (2015). They compared EIH responses in healthy individuals based on CPM levels and discovered that adults with higher CPM were more likely to experience greater EIH, which is consistent with the findings of our previous study (Xu et al., 2022). Thus, descending inhibition might contribute to PPT and PPTol changes following aerobic exercises, which might be modulated by exercise intensity in healthy individuals.

Additionally, low‐ or moderate‐intensity exercise can improve pain tolerance. Vaegter et al. (2016, 2017) investigated the EIH responses during aerobic and resistance exercises and discovered that the PPTol increased following low‐intensity exercises in both healthy and individuals with pain. Hviid et al. (2019) also discovered that changes in the PPTol could be elicited by walking at a relatively low intensity.

The changes in pain tolerance observed during exercise may also be attributed to the improvements in cognitive process (Holmes et al., 2021) related to pain perception in brain areas of “Pain Matrix.” (Salomons et al., 2016) These areas include the prefrontal cortex (Levin et al., 2021), ventral tegmental area (VTA; Liu et al., 2019), amygdala (Kami et al., 2020), nucleus accumbens (NAc), hippocampus, and insular (Villemure et al., 2014). Some rodent studies on neuropathic pain (Kami et al., 2017, 2018) have reported that voluntary exercise activates dopamine neurons in the VTA and gamma‐aminobutyric acid neurons in the NAc shell, which are associated with pain tolerance and emotional aspects, such as anxiety, depression, and fear related to pain perception (Kami et al., 2022; Navratilova et al., 2015). Furthermore, Baiamonte et al. (2017) discovered a negative relationship between changes in PPTol during resistance exercises, HR, and the rating of perceived exertion levels, indicating that exercise fatigue may attenuate EIH effects and reduce tolerance to pain perception.

This study has several limitations. First, the indicators of the pain tests were limited. For instance, adding heat pain detection and tolerance thresholds might provide a more complete description of changes in pain perception. Second, the individuals' pain tolerance thresholds might have been influenced by previous pain experiences and subjective emotional perceptions, leading to a diverse range of responses. Thus, we set strict exclusion criteria to minimize possible bias. Finally, all the participants in this study were female. Considering the potential sex differences in EIH and CPM responses following running exercises, future studies should consider including both male and female participants to provide a comprehensive understanding of these effects.

Thus, aerobic exercise of low to moderate intensity could be an ideal and implementable strategy for preventing and managing various pain conditions, considering the secondary changes in pain perception. However, high‐intensity running revealed no significant advantage in terms of primary or secondary analgesic effects.

## 5 CONCLUSION

Our study revealed that moderate‐ and low‐intensity running induced primary and secondary global hypoalgesia effects and increased CPM responses in females, which may be attributed to the activation of descending pain inhibition, while high‐intensity running only induced limited EIH effects with reduced CPM responses and may elicit descending pain facilitation.

## AUTHOR CONTRIBUTIONS

Zi‐Han Xu and Nan An conceived and designed research, Zi‐Han Xu, Nan An, Jeremy Rui Chang, and Yong‐Long Yang performed experiments, Zi‐Han Xu and Yong‐Long Yang analyzed data. Zi‐Han Xu, Nan An and Jeremy Rui Chang interpreted results of experiments. Zi‐Han Xu prepared Tables and Figures and drafted manuscript. All authors edited and revised manuscript drafts and approved final manuscript.

## FUNDING INFORMATION

This study is self‐funded.

## CONFLICT OF INTEREST STATEMENT

The authors declare no actual or potential conflicts of interest that could influence this study.

## Ethics Statement

This study was approved (2023023H) by the Sports Science Experimental Ethics Committee of Beijing Sport University. Informed consent forms were provided and signed by all participants before participating in this study.

## Supporting information


Tables S1–S8.

## Data Availability

Data available on request from the authors.
