# Supplementary material for: Modulation of pain perceptions following treadmill running with different intensities in females
Source: Physiol Rep. 2023 Sep 25;11(18):e15831. doi: 10.14814/phy2.15831 (PMC10519819; doi:10.14814/phy2.15831)
Supplement: Supplementary file 1 — Tables S1–S8. [file PHY2-11-e15831-s001.docx]

## Supplemental files

Table 2. Changes in PPT-Arm following running exercise (M±SD)

| Measurements | A (n=19) | B(n=21) | C(n=20) | P |
| --- | --- | --- | --- | --- |
| Baseline | 2.31±0.16 | 2.33±0.13 | 2.32±0.17 |  |
| 5min in running^1^ | 2.81±0.21 | 3.09±0.27 | 2.72±0.30 | <0.001^1^ |
| 11min in running^1^ | 3.10±0.33 | 3.45±0.30 | 2.87±0.32 |  |
| 17min in running^1^ | 3.32±0.35 | 3.68±0.31 | 2.98±0.32 |  |
| 23min in running^1^ | 3.46±0.42 | 3.78±0.32 | 3.05±0.32 |  |
| 29min in running^1^ | 3.60±0.34 | 3.80±0.32 | 3.00±0.31 |  |
| 35min in running^1^ | 3.51±0.23 | 3.79±0.29 | 2.95±0.29 |  |
| 5min in follow-up^2^ | 3.38±0.35 | 3.61±0.35 | 2.80±0.28 | <0.001^2^ |
| 10min in follow-up^2^ | 3.27±0.34 | 3.52±0.36 | 2.79±0.31 |  |
| 24h in follow-up^3^ | 2.73±0.23 | 2.83±0.33 | 2.26±0.31 | <0.001^3^ |

All data were presented as mean -/+ standard deviation (M±SD)

^1^: 2-way Repeated measures ANOVA, significant difference was set by p≤0.05.

^2^: 2-way Repeated measures ANOVA, significant difference was set by p≤0.05.

^3^: 1-way ANCOVA, significant difference was set by p≤0.05.

Table 3. Between-group comparison results of PPT-Arm following running exercise

| Measurements | Groups | | Mean Difference | Std. Error | P | 95% CI | |
| --- | --- | --- | --- | --- | --- | --- | --- |
|  |  |  |  |  |  | Lower Bound | Upper Bound |
| 0-35min in running^1^ | A | B | -0.254 | 0.073 | 0.003 | -0.435 | -0.073 |
|  |  | C | 0.317 | 0.074 | <0.001 | 0.134 | 0.500 |
|  | B | C | 0.571 | 0.072 | <0.001 | 0.393 | 0.750 |
| 5-10min in follow-up^2^ | A | B | -0.162 | 0.073 | 0.091 | -0.342 | 0.018 |
|  |  | C | 0.351 | 0.074 | <0.001 | 0.169 | 0.533 |
|  | B | C | 0.513 | 0.072 | <0.001 | 0.336 | 0.691 |
| 24h follow-up^3^ | A | B | -0.103 | 0.092 | 0.800 | -0.329 | 0.123 |
|  |  | C | 0.478 | 0.093 | <0.001 | 0.249 | 0.707 |
|  | B | C | 0.581 | 0.090 | <0.001 | 0.358 | 0.804 |

^1^: 2-way Repeated measures ANOVA, adjusted by Bonferroni. significant difference was set by p≤0.05.

^2^: 2-way Repeated measures ANOVA, adjusted by Bonferroni. significant difference was set by p≤0.05.

^3^: 1-way ANCOVA, adjusted by Bonferroni. significant difference was set by p≤0.05.

Table 4. Changes in PPT-Leg following running exercise (M±SD)

| Measurements | A (n=19) | B(n=21) | C(n=20) | P |
| --- | --- | --- | --- | --- |
| Baseline | 4.40±0.53 | 4.51±0.48 | 4.46±0.48 |  |
| 35min in running^1^ | 5.87±0.74 | 6.21±0.66 | 5.29±0.48 | <0.001^1^ |
| 5min in follow-up^2^ | 5.71±0.71 | 5.95±0.73 | 4.85±0.43 | <0.001^2^ |
| 10min in follow-up^2^ | 5.56±0.67 | 5.88±0.53 | 4.81±0.45 |  |
| 24h in follow-up^3^ | 5.19±0.56 | 5.37±0.50 | 4.21±0.49 | <0.001^3^ |

All data were presented as mean -/+ standard deviation (M±SD)

^1^: 1-way ANCOVA, significant difference was set by p≤0.05.

^2^: 2-way Repeated measures ANOVA, significant difference was set by p≤0.05.

^3^: 1-way ANCOVA, significant difference was set by p≤0.05.

Table 5. Between-group comparison results of PPT-Leg following running exercise

| Measurements | Groups | | Mean Difference | Std. Error | P | 95% CI | |
| --- | --- | --- | --- | --- | --- | --- | --- |
|  |  |  |  |  |  | Lower Bound | Upper Bound |
| 0-35min in running^1^ | A | B | -0.272 | 0.169 | 0.336 | -0.688 | 0.144 |
|  |  | C | 0.614 | 0.170 | 0.002 | 0.195 | 1.034 |
|  | B | C | 0.886 | 0.166 | <0.001 | 0.477 | 1.296 |
| 5-10min in follow-up^2^ | A | B | -0.226 | 0.162 | 0.507 | -0.626 | 0.174 |
|  |  | C | 0.519 | 0.164 | 0.007 | 0.115 | 0.924 |
|  | B | C | 0.745 | 0.160 | <0.001 | 0.351 | 1.140 |
| 24h follow-up^3^ | A | B | -0.119 | 0.135 | >0.999 | -0.453 | 0.214 |
|  |  | C | 1.009 | 0.136 | <0.001 | 0.672 | 1.345 |
|  | B | C | 1.128 | 0.133 | <0.001 | 0.800 | 1.456 |

^1^: 1-way ANCOVA, adjusted by Bonferroni. significant difference was set by p≤0.05.

^2^: 2-way Repeated measures ANOVA, adjusted by Bonferroni. significant difference was set by p≤0.05.

^3^: 1-way ANCOVA, adjusted by Bonferroni. significant difference was set by p≤0.05.

Table 6. Changes in PPTol following running exercise (M±SD)

| Measurements | A (n=19) | B(n=21) | C(n=20) | P |
| --- | --- | --- | --- | --- |
| Baseline | 4.57±0.42 | 4.68±0.49 | 4.61±0.40 | <0.001^1^ |
| 5min in running^1^ | 5.23±0.52 | 5.45±0.61 | 5.21±0.57 |  |
| 11min in running^1^ | 5.57±0.62 | 5.73±0.69 | 5.38±0.74 |  |
| 17min in running^1^ | 5.76±0.64 | 5.96±0.63 | 5.36±0.67 |  |
| 23min in running^1^ | 5.88±0.63 | 6.09±0.69 | 5.50±0.78 |  |
| 29min in running^1^ | 5.97±0.72 | 6.18±0.69 | 5.44±0.66 |  |
| 35min in running^1^ | 5.94±0.69 | 6.23±0.50 | 5.51±0.62 |  |
| 5min in follow-up^2^ | 5.74±0.59 | 6.04±0.51 | 5.40±0.55 | <0.001^2^ |
| 10min in follow-up^2^ | 5.63±0.60 | 5.86±0.56 | 5.22±1.00 |  |
| 24h in follow-up^3^ | 5.27±0.47 | 5.33±0.54 | 4.58±0.57 | <0.001^3^ |

All data were presented as mean -/+ standard deviation (M±SD)

^1^: 2-way Repeated measures ANOVA, significant difference was set by p≤0.05.

^2^: 2-way Repeated measures ANOVA, significant difference was set by p≤0.05.

^3^: 1-way ANCOVA, significant difference was set by p≤0.05.

Table 7. Between-group comparison results of PPTol following running exercise

| Measurements | Groups | | Mean Difference | Std. Error | P | 95% CI | |
| --- | --- | --- | --- | --- | --- | --- | --- |
|  |  |  |  |  |  | Lower Bound | Upper Bound |
| 0-35min in running^1^ | A | B | -0.201 | 0.175 | 0.256 | -0.552 | 0.150 |
|  |  | C | 0.273 | 0.177 | 0.129 | -0.082 | 0.628 |
|  | B | C | 0.474 | 0.173 | 0.008 | 0.128 | 0.821 |
| 5-10min in follow-up^2^ | A | B | -0.208 | 0.160 | 0.199 | -0.529 | 0.113 |
|  |  | C | 0.243 | 0.162 | 0.140 | -0.082 | 0.567 |
|  | B | C | 0.451 | 0.158 | 0.006 | 0.134 | 0.767 |
| 24h follow-up^3^ | A | B | -0.002 | 0.156 | >0.999 | -0.387 | 0.382 |
|  |  | C | 0.711 | 0.157 | <0.001 | 0.323 | 1.098 |
|  | B | C | 0.713 | 0.153 | <0.001 | 0.335 | 1.091 |

^1^: 2-way Repeated measures ANOVA, adjusted by Bonferroni. significant difference was set by p≤0.05.

^2^: 2-way Repeated measures ANOVA, adjusted by Bonferroni. significant difference was set by p≤0.05.

^3^: 1-way ANCOVA, adjusted by Bonferroni. significant difference was set by p≤0.05.

Table 8. Changes in CPM following running exercise (M±SD)

| Measurements | A (n=19) | B(n=21) | C(n=20) | P |
| --- | --- | --- | --- | --- |
| Baseline | 0.70±0.15 | 0.70±0.16 | 0.71±0.17 |  |
| 24h in follow-up^1^ | 0.91±0.25 | 0.90±0.22 | 0.41±0.14 | <0.001^1^ |

All data were presented as mean -/+ standard deviation (M±SD)

^1^: 1-way ANCOVA, significant difference was set by p≤0.05.

Table 9. Between-group comparison results of CPM following running exercise^1^

| Groups | | Mean Difference | Std. Error | Sig. | 95% CI | |
| --- | --- | --- | --- | --- | --- | --- |
|  |  |  |  |  | Lower Bound | Upper Bound |
| A | B | 0.011 | 0.065 | >0.999 | -0.150 | 0.171 |
|  | C | 0.506 | 0.066 | <0.001 | 0.344 | 0.668 |
| B | C | 0.496 | 0.064 | <0.001 | 0.337 | 0.654 |

^1^: 1-way ANCOVA, adjusted by Bonferroni. significant difference was set by p≤0.05.
